# Efficiency and optimization of government service resource allocation in a cloud computing environment

**DOI:** 10.1186/s13677-023-00400-2

**Published:** 2023-02-10

**Authors:** Ya-guang Guo, Qian Yin, Yixiong Wang, Jun Xu, Leqi Zhu

**Affiliations:** 1 School of Management, Hefei University of Technology, 230009 Hefei, Anhui, China; 2 Philosophy and Social Sciences Laboratory of Data Science and Smart Society Governance, Ministry of Education, Hefei, Anhui, China; 3 Anhui Professional & Technical Institute of Athletics, Hefei, China; 4 School of Information Technology, Nanjing Forestry University, Nanjing, China; 5 Ministry of Education Engineering Research Center for Intelligent Decision-Making & Information System Technologies, 230009 Hefei, China; 6 Anhui inter-think tank Data Technology Co., Ltd, Hefei, China

**Keywords:** Government service resources, DEA model, Efficiency, Cloud computing resources

## Abstract

According to the connotation and structure of government service resources, data of government service resources in L city from 2019 to 2021 are used to calculate the efficiency of government service resource allocation in each county and region in different periods, particularly by adding the government cloud platform and cloud computing resources to the government service resource data and applying the data envelopment analysis (DEA) method, which has practical significance for the development and innovation of government services. On this basis, patterns and evolutionary trends of government service resource allocation efficiency in each region during the study period are analyzed and discussed. Results are as follows. *i*) Overall efficiency level in the allocation of government service resources in L city is not high, showing an increasing annual trend among the high and low staggering. *ii*) Relative difference of allocation efficiency of government service resources is a common phenomenon of regional development, the existence and evolution of which are the direct or indirect influence and reflection of various aspects, such as economic strength and reform effort. *iii*) Data analysis for the specific points indicates that increased input does not necessarily lead to increased efficiency, some indicators have insufficient input or redundant output. Therefore, optimization of the physical, human, and financial resource allocation methods; and the intelligent online processing of government services achieved by the adoption of government cloud platform and cloud computing resources are the current objective choices to realize maximum efficiency in the allocation of government service resources.

## Introduction

Government service resources are the bases for building a service-oriented government and improving the capacity of government services. However, building a high-quality service system requires sufficient resource input and also relies on efficient service resource allocation management. Government service resources account for the largest share of the overall government service resources, and the efficient, fair, and reasonable allocation of these resources to various users in need is immensely challenging.

A strong demand for e-government services has been raised from the reform of the administrative approval system and construction of service-oriented government. E-government has become an enabler of service-oriented and clean government, specifically with its advantages of efficiency and transparency [1]. Moreover, the development and application of cloud computing technology has further promoted the development of e-government, which provides the public and enterprises with efficient and intelligent government services. Such services are provided by integrating the application and data resources, such as government approval process and business information base, performing calculations and multi-dimensional analysis, and realizing the three major computing capabilities of background perception, intelligent aggregation, and interactive presentation (i.e., government cloud). The government cloud [2–4] cannot be implemented without the support of government service resources (e.g., infrastructure construction, support software, application systems, and information resources) and cloud computing resources. The government service cloud provides a new option for government service resource input, thereby giving rise to the redistribution and reuse of government service resources.

Current studies have found that input (not output) and scale (not performance) are emphasized in the allocation of government service resources. Considering that regional differences happen in the efficiency of government service resource utilization, and the same government service resource input may bring different levels of output, this paper takes the efficiency of government service resource allocation as the research object. On the basis of the current difference of government service resource allocation in L city, Anhui Province, indicator models are first established from three aspects: human, financial, and material (including government cloud computing resources). From the perspectives of inputs and outputs and through the DEAP efficiency analysis model, the efficiency of government service resource allocation is evaluated through data envelopment analysis [5–7]. Moreover, the influence of resource inputs and structure on the comprehensive efficiency, pure technical efficiency, and scale efficiency of the input impact is analyzed. Lastly, the research findings are summarized and policy recommendations are proposed [8–10].

## Related studies

Cloud computing is an organization, configuration and usage mode with computing resources such as high-performance computing and high-capacity storage. It distributes computing tasks on the resource pool composed of a large number of computers. In this computing mode, the computing capacity, storage capacity, load capacity and other issues of the terminal are no longer factors limiting users, which greatly improves the utilization of information resources, significantly reduces operating costs and improves operating efficiency [11, 12]. Cloud computing environment is a typical distributed computing environment, which allocates task scheduling to the best resources for execution. In the cloud computing resource environment, resources are heterogeneous and extensible and can be freely combined to provide services for different tasks. The task scheduling algorithm with good performance can optimize the quality of service and improve resource utilization, reliability, execution cost, etc. With the development of intelligent terminals and new network applications, real-time and effective massive computing of terminal devices and energy issues have become key areas of concern to improve the efficiency of cloud computing, although cloud computing has strong computing power. Relevant research mainly focuses on edge computing, energy management, task unloading, etc. Mobile terminal devices that are computing intensive or sensitive to delay can offload computing tasks to edge servers for computing to reasonably complete task scheduling [13–15], energy management and resource allocation  [16, 17], thereby improving the efficiency of the cloud computing environment [18–20].

In relation to government services in the cloud computing environment, with the continuous evolution of the COVID-19 pandemic, governments at all levels have increasingly invested in the field of public health, which has led to a shrinkage in other public expenditures. Therefore, using cloud computing technology to establish the government cloud services platform can realize the sharing of government resources among government departments and government service customers, thereby helping to avoid the repetition of establishing multiple platforms and saving funds. At the same time, the continuous establishment of service-oriented government and the continuous optimization of public administration processes also contribute to the establishment of e-government on government cloud services platforms.

Researchers in this field have long realized the necessity of migrating e-government to cloud computing. While investigating cloud computing technology, they have attached considerable importance to the application of cloud computing technology in e-government, especially the management and application of government service resources on government cloud services platforms. Therefore, in existing government service resource management, a broader view of human, finance, and material differences from the past should be adopted and understood in e-government and government cloud services. Here, cloud computing resources, as the medium and foundation of e-government establishment, also need to be regarded as a fundamental aspect of allocating and managing government service resources together with human, financial, and material. In this case, how to achieve a rational allocation of government service resources by integrating cloud computing resources in the whole process of the e-government service process and realize the integration and sharing of various government services across departments to improve the service capabilities of a service-oriented and transparent government will be a problem that has to be faced.

At present, the relevant studies have focused on the efficiency of resource allocation in medical resources [20], higher education [21], scientific and technological resources [22], environmental protection investment [23], and urban management [20, 24]. The definition of the corresponding resources is mainly developed in three dimensions: human, financial, and physical resources. According to related research, government service resources can also be defined in three dimensions: human, financial, and material resources. However, the difference lies in that, owing to the demand and application of “government cloud,” a previous study has indicated that the relevant indicator of “government cloud” resources should be added to the physical resources (i.e., indicators of government affairs information resources).

Related research has shown that developed countries mainly adopt the measures and solutions of market-led and moderate government participation [25] in evaluating the effective allocation of government service resources. Therefore, their allocation evaluation is mainly based on such aspects as cost-benefit method and price mechanism. The measures taken by developing countries are mainly in the form of strengthening infrastructure, increasing investment, and training [26–28]. In China, studies have been focused on the allocation efficiency and scale in terms of human and financial resources. In addition, the effectiveness of resource allocation has been evaluated and analyzed by neural networks and hierarchical analyses, among others. In terms of resource allocation efficiency, extensive studies on resource allocation efficiency in other fields. For science and technology, agriculture, urban public infrastructure [29–31], and medical care. Efficient research methods are used for research and improvement to perform investigation and efficiency evaluation for resource allocation in various fields.

Government service resources are similar to other fields in terms of the principle and composition of resource allocation, covering human, finance, and material indicators. The existing literature has revealed that only a few scholars have studied the overall efficiency of government service resource allocation. Moreover, the adoption of functional analysis and other econometric methods in existing studies has provided substantial empirical evidence on the efficiency of government service resource allocation and efficiency impact factors. However, the following problems remain. First, “cloud computing” resource data is lack considering the application needs of “government cloud.” Second, output is measured using a relatively single indicator. Third, the function approach cannot solve the endogeneity problem generated by resource inputs, and the results cannot reflect the causal effect. To solve the preceding problems, this paper added “cloud computing” resource indicators to meet the new development needs of “government cloud.” Meanwhile, input and output indicator systems are constructed from multiple dimensions. The DEA efficiency model method is adopted to avoid pre-determined production functions, and deep data patterns of input-output indicators for government service resources are discovered. This result provides an objective basis for optimizing the allocation of government service resources and promoting the maximum efficiency of government service resources.

## Research Design

### Allocation efficiency measurement using the DEA model

The DEA method [6–10] is widely used for the efficiency evaluation of integrated services under multi-conditional constraints owing to its advantages (e.g., no need to pre-determine specific generating functions, ability to handle multiple input and output problems). Different from subjective evaluation methods, such as hierarchical and weight analyses, DEA is an objective efficiency evaluation method. Allocation of government service resources studied in this paper is characterized by multiple inputs and outputs, and each decision unit (different government service halls) has the same objectives and tasks. These aspects are consistent with the principles of the input-output index setting in the DEA model.

By referring to the relevant research models, the DEA method is used to test the efficiency of allocation for government service resources. The common models of DEA mainly include CCR model and BCC model. The difference between them is the assumption that the former assumes constant returns to scale (CRS) and the latter assumes variable returns to scale (VRS). On the basis of the assumption of variable returns to scale (BCC), the decision-making unit can expand the scale benefit of output by increasing the input of equal proportion elements and the efficiency of each decision unit is accounted from the perspective of output. The assumption is that each county (district) government service resource is ${DMU}_K$ with *m* *DMU*, and each *DMU* has *i* and *j* types of inputs and outputs, respectively. In general, few inputs or large outputs are considerably preferred.

Input and output vectors are set as $X_k=(x_{1k}, x_{2k}, x_{3k}, \dots , x_{ik})$ and $Y_k={(y_{1k}, y_{2k}, y_{3k}, \dots , y_{jk})}^T$ , respectively;

1 $$\begin{aligned} D_{C^2R}=\left\{ \begin{array}{r} min\theta \\ s_.t_.\sum \limits _{j=1}^n x_j\lambda _j+s^-=\theta x_k\\ \sum \limits _{j=1}^n y_j\lambda _j-s^+=\theta y_k\\ \lambda _j \ge 0\\ s^+ \ge 0, s^- \ge 0\\ \end{array} \right. \end{aligned}$$

where $\theta$ efers to the degree of efficient use of inputs relative to outputs, which is the efficiency of the decision unit; $\theta =1$ indicates that the decision unit is an efficiency frontier and technically efficient decision unit; $\theta <1$ indicates a technical loss; and $s^+$ and $s^-$ are slack variables indicating the ${DMU}_K$ input redundancy and output deficiency, respectively, relative to the frontier surface. Given the continuity of the input of government service resources and the service itself, the input and output have formed a stable relationship, and thus, the time lag effect will not be considered.

### Analysis of factors affecting the government service resource allocation

After the central government promotes the streamlining of administration, decentralization of authority, and the reform of administrative review and approval, local governments have to deal with more administrative affairs. Whether the allocation of local government information resources is reasonable and effective directly determines the interests of the public and social equity within the administrative region.

In this paper, the input resources are divided into three aspects: human, financial, and material resources (material resources include information system and cloud computing resources), and the output resources are measured in terms of the quantity and quality of the public services accomplished. The efficiency analysis software is then used to analyze the factors affecting the local government information resource allocation.

## Establishment of the allocation efficiency evaluation model of government service resources

### Data sources

In accordance with the application requirements of e-government, computing resources of the “government cloud” are uniformly classified as physical resources of government service resources in a broad sense. Data were obtained from the annual report of information disclosure of the L city government and survey statistics of 3 districts, 4 counties, and 1 city under the city’s jurisdiction (8 regions). The study period was 2019-2021, and data were processed and described as follows. (1) All data were collected and sorted according to the spatial partition of data. (2) Cloud computing resource data [32, 33] were performed for metrics design.

### Construction of index systems

Before establishing the evaluation model, the decision unit, input indicators, and output indicators should be set first. This paper will divide input resources according to the existing research basis in three dimensions, namely, human, financial, and physical resources, in which physical resources contain information system and cloud computing resources. Combined with the business characteristics of government services, input indicators are set as follows: number of window practitioners is set as I1; number of helpers and agents, I2; number of management and logistic personnel, I3; department windows, I4; overall windows, I5; number of self-service machines and other smart devices, I6; number of online processing channels, I7; annual financial account expenses, I8; number of cloud hosts, I9; physical number of hosts, I10; Internet bandwidth, I11; and number of cloud hard disks, I12. For the tasks and goals of the construction of integrated government services, this paper constructs the output index system of government service resources from four dimensions: annual processing matters, processing channels, public satisfaction, and processing effects. Output indicators are set as follows: number of trans-provincial service items is set as O1; total number of annual items, O2; online service rate, O3; good and bad rating satisfaction rate, O4; and active evaluation rate, O5. The definitions and sources of the selected variables are shown in Table 1. The descriptive statistics are presented in Table 2.

**Table 1:** Input and output index system of the government service resources

Types	Dimensions	Indicator layers	Units	Data sources
	Human resources	Window practitioners (I1)	Number	
		Helpers and agents (I2)	Number	
		Management and logistics personnel (I3)	Number	Annual work report of each unit
	Material resources	Departmental windows (I4)	Number	
		Overall windows (I5)	Number	
Input indicators		Self-service machines and other smart devices (I6)	Set	
		Online service channels (I7)	Number	
	Financial resources	Annual financial account expenses (I8)	10,000 yuan	Annual final accounts report of each unit
	Cloud computing resources	Cloud hosts (I9)	Number	
		Physical hosts (I10)	Number	
		Internet bandwidth (I11)	G	Statistics obtained from survey questionnaire
		Cloud hard disks(I12)	T	
	Service Items	Service items in Trans-provincial Netcom(O1)	Number	
Output indicators		Total service items (O2)	Number	
	Service channels	Online service rate (O3)	%	Statistics obtained from survey questionnaire
	Service effects	Good and bad rating satisfaction rate (O4)	%	Public information in the government service network
		Active evaluation rate (O5)	%	

**Table 2:** Descriptive statistics of input and output variables of government service resources

Types	Variables	Number of cases	Minimum	Maximum	Average value	Standard deviation
Input indicators	I1	27	70	460	174.3	120.1
	I2	27	1	23	5.5185	6.756
	I3	27	8	38	19.18	8
	I4	27	1	295	65.22	87.03
	I5	27	0	26	4.92	6.78
	I6	27	0	22	9.03	6.69
	I7	27	3	4	3.667	0.48
	I8	27	139.9	2855.68	713.09	688.6
	I9	27	3	950	196.85	210
	I10	27	1	10	5.58	3.92
	I11	27	0.3	3	1.32	0.85
	I12	27	1	755	182.51	165.64
Output indicators	O1	27	1	132	42.62	36.86
	O2	27	123561	28270318	2391886	6986171
	O3	27	0.01	0.48	0.1	0.11
	O4	27	0.99	1	0.991	0.003
	O5	27	0.01	1	0.289	0.376

### Correlation analysis of input-output indicators

The principles of constructing the indicator system [34] indicate that the correlation between input and output indicators cannot be significantly high, while the correlation between the input and output indicators should be high. SPSS is used to test the correlation Pearson coefficients of the input and output indicators of government service resource allocation. The results (Table 3) show that all input and output indicators exert positive correlations. Except for the correlations between I4 and O5 and that between I12 and O3, which are not significant at the 10% level, all the others exert significant positive correlations at the 5% level and meet the requirements of DEA for variables.

**Table 3:** Results of the Pearson correlation coefficient analysis for the input and output indicators

Output												
Input	I1	I2	I3	I4	I5	I6	I7	I8	I9	I10	I11	I12
O1	0.242***	0.472***	0.224***	0.073	0.180**	0.266***	0.259***	0.603***	0.288***	0.397***	0.131**	0.206***
O2	0.700***	0.258***	0.151**	0.779***	0.175**	0.232***	0.043	0.191**	0.809***	0.537***	0.233***	0.790***
O3	0.115**	0.315***	0.375***	0.212***	0.253***	0.177**	0.226***	0.360***	0.223***	0.241***	0.226***	0.063
O4	0.164**	0.136**	0.323***	0.108**	0.189**	0.151**	0.138**	0.279***	0.193**	0.136**	0.243***	0.135**
O5	0.177**	0.402***	0.125**	0.014	0.119**	0.401***	0.213***	0.201***	0.278***	0.165**	0.123**	0.181**

Note: *** and ** indicate significant correlation at the 1% and 5% levels (two-tailed), respectively

## Empirical analysis

This paper selects the government service resources (including cloud computing resources) of L city and its three districts, four counties, and one city under its jurisdiction from 2019 to 2021. DEAP 2.1 software is applied to derive the overall effectiveness, technical effectiveness, returns to scale, and each input and output index of its resource allocation efficiency.

**Table 4:** Allocation efficiency of government service resources in L city and counties under its jurisdiction, 2019-2021

District	2019			2020			2021		
	EE	TE	SE	EE	TE	SE	EE	TE	SE
L City	0.305	0.581	0.525	1	1	1	1	1	1
Funan County	0.063	1	0.063	0.226	0.788	0.288	0.801	0.88	0.91
Jieshou City	0.67	1	0.67	0.794	0.891	0.891	0.812	0.891	0.911
Linquan County	0.567	0.891	0.636	0.794	0.891	0.891	0.803	0.881	0.912
Taihe County	0.254	0.809	0.314	0.136	0.779	0.174	0.798	0.891	0.896
Yingdong District	0.053	0.867	0.061	0.362	0.786	0.461	0.799	0.887	0.901
Yingquan District	0.275	0.891	0.309	0.365	0.814	0.448	0.794	0.891	0.891
Yingshang County	0.795	0.891	0.892	0.794	0.891	0.891	0.804	0.892	0.901
Yingzhou District	0.442	0.891	0.496	0.782	0.891	0.878	0.794	0.891	0.891

### Overall input and output efficiency for government service resources allocation

Table 4 shows the input-output efficiency values of government service resources in L city for 2019-2021, where EE is comprehensive efficiency, TE is pure technical efficiency, SE is scale efficiency, and EE = TE * SE. TE reflects the degree of effective use of available technology in the decision unit at a given input scale. SE refers to the matching degree of infrastructure inputs to outputs. Comprehensive efficiency reflects the input-output level of industry innovation with the available technologies and the technical level of a decision-making unit.

Effectiveness analysis: L City in 2020 and 2021, the evaluation result is $\theta$=1 and the $s^{+}$ and $s^{-}$ are 0, so the data in these two years are “DEA is strong and effective”.

**Fig. 1:**
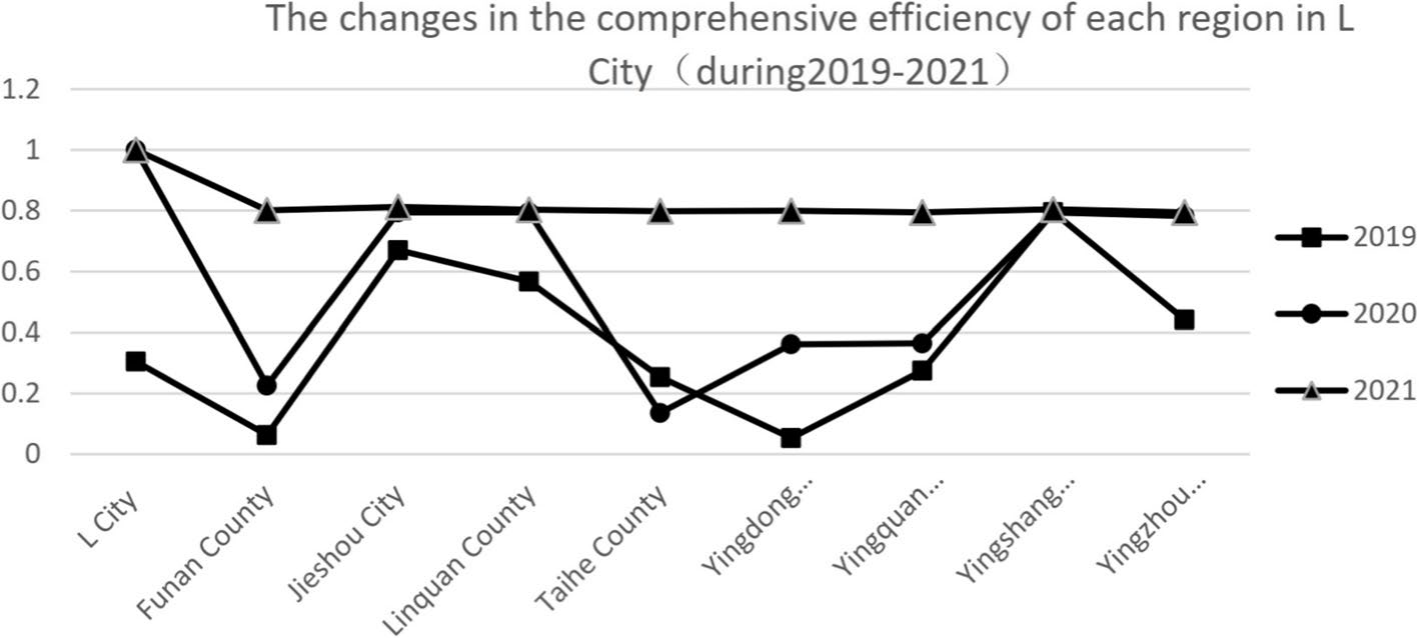
The changes in the comprehensive efficiency of each region

Table 4 shows that the overall efficiency of government service resource allocation in L city is improving, with the average comprehensive efficiency at 0.596, TE of 0.876, and SE of 0.670. Given the trend of change, efficiency was lowest in 2019, with average comprehensive efficiency of 0.380, TE of 0.869, and SE of 0.440. With the implementation of the e-government and “one network, one door, one time” reforms, efficiency values increased in 2020 and 2021, and reached at least 0.8 in 2021. Since 2019, local governments have gradually realized problems in the service-oriented resource allocation method. As a result, they have begun to improve the scale of government service input and the efficiency of financial input to promote the even allocation of government services. Taking the comprehensive efficiency that reflects the overall efficiency level as an example, the changes in the comprehensive efficiency of each region are shown in Fig. 1.

**Table 5:** Analysis of dynamic changes in resource allocation efficiency

Changes in technological progress					
Year	tfp	tc	tec	sec	ptec
2019	1.505	0.648	1.057	1.425	0.975
2020	1.156	1.156	0.992	1.165	1.337
2021	1.319	0.866	1.024	1.289	1.142

### Analysis of dynamic changes in resource allocation efficiency

Based on DEA Malmquist index analysis method, as shown in Table 5, the efficiency of government service resource allocation in each region from 2019 to 2021 is calculated and decomposed into comprehensive technical efficiency progress (tfp), technical change (tc), technical efficiency change (tec), scale efficiency change (sec), and pure technical efficiency change (ptec).

The comprehensive technical efficiency will steadily improve from 2019 to 2021, but the growth rate will be unstable with the highest annual growth rate of 50.5% and the lowest annual growth rate of 15.6%. Secondly, resource allocation efficiency is mainly affected by changes in technical efficiency. The technical progress is less than the change of technical efficiency, which indicates that the improvement of resource allocation efficiency is mainly affected by the change of technical efficiency. The continuous improvement of government resource allocation capability and level drives the improvement of resource allocation efficiency.

### Analysis of the spatiotemporal distribution of input-output efficiency

From the analysis in Table 4, the efficiency of allocation of government service resources generally presents a state of “high in the urban area and the eastern and western regions of City L and low in the north and south regions,” and regional differences lead directly to differences in the efficiency of government service resources. For example, Yingshang County, in the eastern region with the highest average comprehensive efficiency, is the most populous county in the city. An analysis of its government service resource data shows that the county’s government investment in the past three years has averaged about 4.36 million yuan, while the city’s government investment has averaged 7.13 million yuan per year in the past three years. At the same time, the number of employees and service windows are also below the city’s average. However, because of its large population, the number of government services accomplished throughout the county year is above the average, resulting in higher overall efficiency of its government services.

Taihe County, in the northern region with the lowest average comprehensive efficiency, is an area with a relatively high GDP in the city. An analysis of its government service resource data show that in the past three years, with the growth of GDP, the county has continued to increase the government service resource input. Despite the increase in the number of public services accomplished, which is an indicator of the output, the overall efficiency is still at a low level throughout the year. The structure and utilization of government service resources need to be studied and planned scientifically.

The urban area of City L ranks second in terms of overall efficiency, second only to Yingshang County, a county with a large population. With the acceleration of urbanization, it is the area with the largest government service resources and the highest number of services accomplished in the city. Government service and cloud computing resources are invested in the urban area, and to save resources, some counties and districts share the cloud computing resources with the urban area. Thus, the resource allocation efficiency of the urban area ranks only second.

### Analysis of redundant input and insufficient output

Among the analysis results obtained in the DEAP software, the slack movement value is 0, indicating relatively reasonable resource allocation and the absence of redundant input and insufficient output. The slack movement value is negative on the input indicator, which represents the gap between the target value and the original value and indicates excessive input and redundant output. The slack movement value is negative on the output indicator, representing insufficient output.

The results show that for the urban area of City L, in 2019, the four input indicators, including the number of public-facing employees, the number of service windows, annual financial settlement expenditure, and the number of cloud hard disks, have redundant input, while the number of inter-provincial government services has insufficient output. In Funan County, in 2019, the number of public-facing employees and the number of cloud hard disks have redundant input, while the number of inter-provincial government services has insufficient output. In 2020, the number of cloud hard disks had redundant input, and the number of services accomplished had insufficient output. In Taihe County, in 2019, the number of service windows, annual financial settlement expenditure, and the number of cloud hard disks have redundant input. In 2020, the number of service windows, annual financial settlement expenditure, and cloud hard disks had redundant input, while the number of inter-provincial government services had insufficient output. In Yingdong District, in 2019, the number of inter-provincial government services had insufficient output. In 2020, the number of management and logistics personnel, the number of cloud hosts, and the number of cloud hard disks had redundant input. In 2021, the number of public-facing employees will have redundant input. In Yingquan District, the number of management and logistics personnel, the number of cloud hosts, and the number of cloud hard disks have redundant input. In the rest of the years, the slack movement value of each region is 0, indicating relatively reasonable input and output and the absence of redundancy or insufficiency.

## Conclusions and recommendations

Allocation efficiency of government service resources at the municipal and county levels in L city for 2019-2021 is analyzed using the DEA method. This paper determined the following common problems.

(1) The overall comprehensive efficiency of the input and output of government service resource allocation in L city is not high. One of the reasons is the unreasonable allocation of input resources for government service. That is, resource utilization and transformation are redundant, making resources underutilized.

(2) Blindly expanding resource input cannot improve the efficiency of resource allocation for government services. Instead, it may lead to increased cost and low efficiency of resource security, resulting in wastage of the additional portion of resources invested. For example, financial statement expenses are considerably high in Taihe County in 2019 and 2020 (¥13,246,300 and ¥28,556,800, respectively). However, the advantage of scale cannot be developed owing to unreasonable scheduling and distribution.

(3) Because no in-depth research on the underlying data indicators of government service resources has been conducted, identifying internal problems in resource allocation is impossible. The lack of scientific and in-depth research on whether a certain indicator indicates redundancy or insufficiency will result in the misjudgment of whether the internal allocation of government service resources is reasonable, and the traditional blind investment requires further scientific research.

Given the preceding problems, this paper proposes the following countermeasures and suggestions.

(1) Improve the scientificity of government service resource allocation to enhance resource utilization

Government service resources should be modeled, analyzed, and mined using statistical models, algorithms, and technologies, such as intelligent analysis and forecasting. The reason is to deeply discover the endogenous correlation of input indicators and its causal relationship with output indicators. Hence, scientific decision-making basis can be provided for the government’s rational deployment of inputs and resources.

(2) Introduce a tripartite monitoring mechanism to enhance the utilization of government service resources

A multi-level regulatory mechanism should be established to supervise the allocation and utilization of government service resources, such as controlling personnel input and capital budget, through the government, industry, and people, to substantially avoid abuse and wastage of resources.

(3) Use the government cloud platform to increase resource sharing and security in poor areas and populations to improve the utilization of cloud computing resources

Establish a sharing mechanism of redundant cloud computing resources to solve the problem of redundant input indicators of cloud computing resources. Widely promote internet+e-government to make government services are not limited by geographical space, improve the utilization of resources and create an efficient government. Secondly, we will build a conservation-oriented government through reasonable integration of government cloud computing resources.

## Data Availability

The data has been gathered from research papers and articles.

## Abbreviations

DEA: Data Envelopment Analysis
CCR: A. Charnes & W. W. Cooper & E. Rhodes
DMU: Decision Making Units
COVID-19: Corona Virus Disease 2019
